# Effect of fluoride dentifrice and casein phosphopeptide-amorphous calcium phosphate cream with and without fluoride in preventing enamel demineralization in a pH cyclic study

**DOI:** 10.1590/1678-7757-2016-0559

**Published:** 2017

**Authors:** Priscila de Pinto Sinfiteli, Thereza Christina Lopes Coutinho, Patrícia Regina Almeida de Oliveira, Wesley Felisberto Vasques, Leandra Matos Azevedo, André Maues Brabo Pereira, Monica Almeida Tostes

**Affiliations:** 1 Universidade Federal Fluminense, Faculdade de Odontologia, Niterói, RJ, Brasil.; 2 Universidade Federal Fluminense, Faculdade de Odontologia, Departamento de Odontopediatria, Niterói, RJ, Brasil.; 3 Universidade Federal Fluminense, Faculdade de Engenharia, Niterói, RJ, Brasil.

**Keywords:** Hardness tests, Calcium fluoride, Enamel, Dental caries

## Abstract

Casein phosphopeptide-amorphous calcium phosphate (CPP-ACP) complexes are anticariogenic and capable of remineralizing the early stages of enamel lesions. The use of fluoride prevents dental decay and the association of CPP-ACP with fluoride can increase remineralization.

**Objective:**

To evaluate the effect of CPP-ACP and CPP-ACPF creams associated with a fluoride dentifrice to prevent enamel demineralization in a pH cyclic model.

**Material and Methods:**

Previously selected by surface microhardness (SH) analysis, human enamel blocks (n = 56) were submitted to daily treatment with dentifrice in a pH-cycling model. The enamel blocks were divided into four groups; G1: Crest™ Cavity Protection - Procter & Gamble (1,100 ppmF of NaF); G2: Crest™ +MI Paste (MP) - Recaldent™ GC Corporation Tokyo, Japan); G3: Crest™ + MI Paste Plus (MPP) - Recaldent™ 900 ppm as NaF, GC Corporation Tokyo, Japan), and G4: control, saliva. Specimens were soaked alternatively in a demineralizing solution and in artificial saliva for 5 d. The fluoride dentifrice, with proportion of 1:3 (w/w), was applied three times for 60 s after the remineralization period. The undiluted MP and MPP creams were applied for 3 m/d. After cycling, SH was re-measured and cross section microhardness measurements were taken.

**Results:**

The SH values observed for the groups G3 (257±70), G1 (205±70), and G2 (208±84) differed from the G4 group (98±110) (one-way ANOVA and Tukey's *post hoc* test). There were no differences between the groups G1xG2, G2xG3, and G1xG3 for demineralization inhibition. The percentage of volume mineral showed that, when applied with fluoride dentifrice, MPP was the most effective in preventing enamel demineralization at 50 µ from the outer enamel surface (Kruskal-Wallis and Mann Whitney p<0.05).

**Conclusion:**

Fluoride dentifrice associated with CPP-ACPF inhibited subsurface enamel demineralization.

## Introduction

The complex casein phospho-peptide-amorphous calcium phosphate (CPP-ACP) comes from a milk protein called casein. CPP-ACP represents an alternative-remineralizing agent, capable of stabilizing calcium phosphate, maintaining the supersaturation of these ions in the oral environment. Amorphous calcium phosphate (ACP) favors the proximity between calcium and phosphate ions in an amorphous phase. They can enhance remineralization, decrease demineralization or even both in an acid challenge to teeth surfaces^4^ ^,^ ^17^ ^,^ ^18^.

*In vitro* ^1^ ^,^ ^5^ ^,^ ^7^ ^,^ ^10^ ^,^ ^12^ ^−^ ^14^ ^,^ ^16^ ^,^ ^17^ ^,^ ^23^ and *in vivo* ^3^ ^,^ ^19^ ^,^ ^20^ studies have been carried out to understand the association of CPP-ACP with fluoride toothpaste; however, there is little knowledge on whether this combination improves remineralization. Furthermore, some studies have shown that there was no additional effect of CPP-ACP on the remineralization of artificial caries when used as a supplement to the regular fluoride toothpaste^10^ ^,^ ^16^. Meyer-Lueckel, et al.^11^ (2015) compared the remineralizing effects induced by the application of casein phosphopeptide-stabilized amorphous calcium phosphate complexes (CPP-ACP containing cream without fluoride) after the use of fluoride toothpaste in an *in situ* model. They concluded that the additional use of a CPP-ACP containing cream seems to be less efficient in remineralizing caries lesions than in the prolonged application of fluoride toothpaste. In contrast, Reynolds, et al.^18^ (2008), also using an *in situ* caries model, showed that the amount of fluoride incorporated into the lesions was significantly higher for 2% CPP-ACP with 1,100 ppm F dentifrice when compared to that containing 1,100 ppm F alone. In addition, other authors have shown that there was a higher increase in remineralization and a decrease in lesion depth when combining the two^12^.

Considering there are few reports on the effectiveness of CPP-ACP associated with fluoride dentifrice, it seems relevant to investigate its performance regarding the enamel surface and remineralization throughout the lesion.

The surface hardness test (SH) used in some studies has shown to be sensitive enough to detect the early stages of enamel demineralization, occurring at the enamel surface, but not able to measure lesion depth^1^ ^,^ ^8^ ^,^ ^14^ ^,^ ^22^ ^,^ ^23^. Thus, we used cross section hardness (CSH) to evaluate lesion depth. The technique is well established for enamel and can be used with enamel block lesions^2^ ^,^ ^6^ ^,^ ^9^.

The aim of this study is to evaluate the effect of associating CPP-ACP and fluoride toothpaste in enamel remineralization, using SH and CSH. To do so, we tested the null hypothesis that, in a pH cycling model system, remineralization treatments, such as fluoride dentifrice, MI Paste (MP), and MI Paste Plus (MPP), when associated with fluoride dentifrice, are not more efficient to reduce lesion progression compared to a placebo (artificial saliva).

## Material and Methods

### Sample preparation

In this study, 20 human third molars, which had been extracted for surgical reasons, were used. The study was approved by the Ethical Committee of Medical Science, Fluminense Federal University (Protocol number 393000/13). The sample size was performed assuming 80% of power to detect the difference between the treatments at a 5% significance level (http://www.openepi.com). A minimum of 12 was required. The samples were free of caries and fluorotic or hypomineralized lesions and any other visible defects. The teeth were stored in thymol 0.1% during the sample preparation process. A total of 80 enamel blocks were obtained from the buccal and lingual surfaces of 20 third human molars. After embedding the blocks in acrylic resin, the buccal surfaces of the enamel specimens (2×2×2 mm) were ground with SiC paper (400, 600 and 1,200 grits) (Struers S/A, Struer, Denmark) to obtain fiat surfaces. Then, the specimens were polished using a 1 μm diamond polishing suspension with a polishing cloth (Arotec Ind. & Com., Cotia, SP, Brazil). The baseline surface microhardness (SH) of all specimens was measured using a microhardness tester (Micromet 2001, Buehler,, IL, USA) with a Knoop diamond indenter under a 50 g load for 15 s. Five indentations, spaced 50 µ apart, were made and the mean values of enamel surface hardness were calculated (SH baseline=SH_0_). After SH measurements, 56 enamel blocks were selected with a Knoop hardness number (KHN) ranging from 275.72 to 369.72. The enamel blocks were distributed into 4 groups of 14 blocks each: Group 1 - Crest ^TM^ Cavity Protection (FD – 1,100 ppm F of NaF) - Procter & Gamble); Group 2 - Crest™ + MI Paste (MP) - Recaldent™ (GC Corporation Tokyo, Japan); Group 3 - Crest™ + MI Paste Plus (MPP) - Recaldent™ 900 ppm as NaF (GC Corporation Tokyo, Japan); and Group 4 - saliva, used for negative control.

### pH cycling and treatment with dentifrices

**Figure 1:**
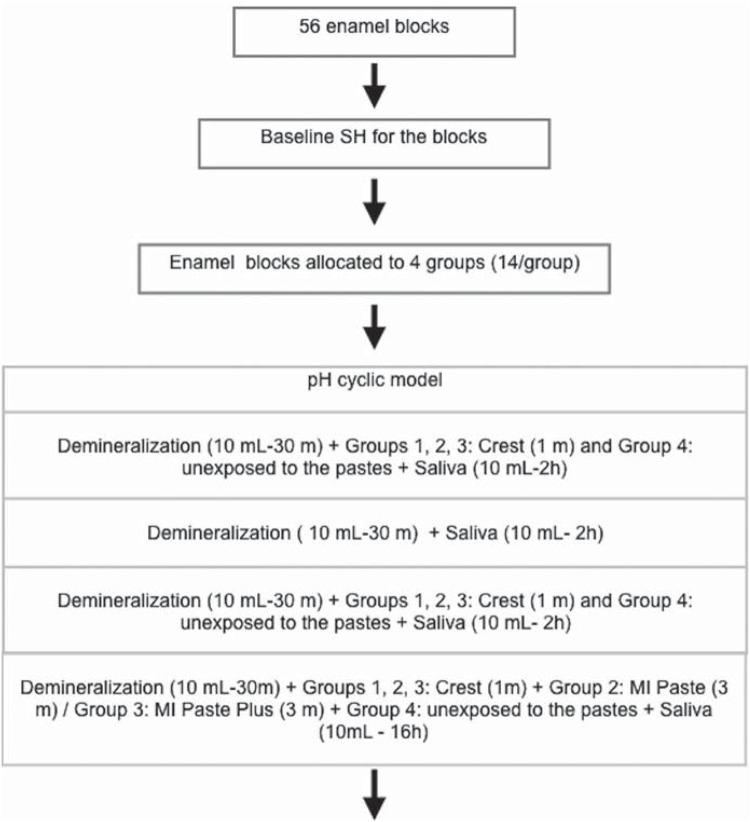
Flow chart illustrating the study protocol

The enamel blocks were submitted to pH cycles for 5 d, at 37°C. After sonication and the rinsing with distilled water, the specimens were immersed separately in 10 mL of demineralizing solution [2 Mm Ca (Ca (NO_3_)_2_, 2 Mm PO_4_ (KH_2_ PO_4_) and 75 Mm of acetate at 4.3 pH]^22^ according to the protocol in Figure 1. The artificial saliva was renewed daily and comprised 0.67 g/L NaCl; 0.1168 g/L CaCl_2_; 8 g/L CMC; 0.0408 g/L MgCl_2_; 0.96 g/KCL; 1 g/L C_8_H_8_O_3_; 24 g/L C6H_14_O_6_; 964,938 ml/L H_2_O; 0.274 g/L KH_2_ PO_4_ ^14^.

At each transfer between different solutions, the enamel specimens were rinsed in distilled water for 1 m at 37°C. The treatment was applied in slurry in a proportion of 1:3 of deionized water for 60 s, three times a day, in the groups. A standardized volume (0.15 mL) was applied in each sample. The MP (Group 2) and MPP (Group 3) formulas were used undiluted (0.03 g) for 3 m/d. Negative control (Group 4) remained unexposed to the pastes. The treatments were carried out before the first, the second and the third demineralization cycles. After the last demineralization challenge, the enamel specimens were rinsed in distilled water for 1 m, and then immersed in 10 mL of artificial saliva. The solutions were changed every day (Figure 1).

**Figure 2:**
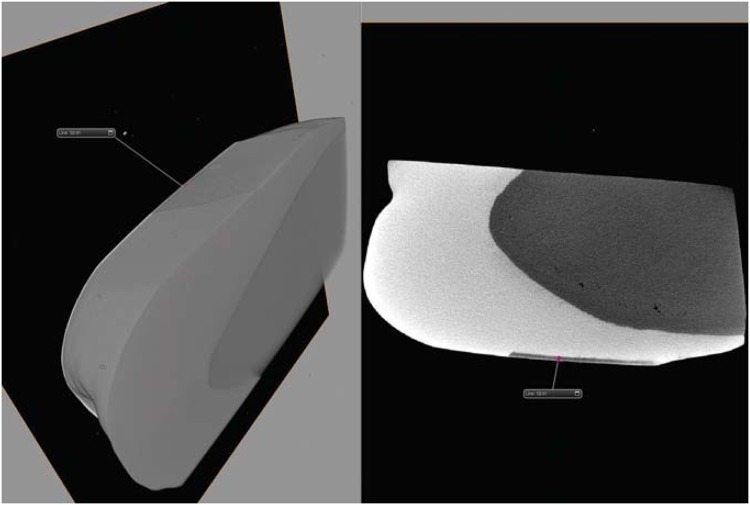
Typical 3D images of enamel blocks in the control group after pH cycling

The lesion depth value formed with this protocol was tested previously. The three enamel blocks were submitted to pH cycles as described for control group. After, the blocks were scanned with micro-CT (ZEISS Xradia 510 Versa). Three-D image analysis software (AVIZO) was used for visualization. The formed lesion depth was of 59.81 µm (Figure 2).

### Surface hardness (SH)

Pre and post-treatment measurements (SH_0_ and SH_1_, respectively) were conducted with the same static load and time used in baseline measurements. Five indentations with a space of 100 μm from the baseline indentations were made with a Knoop diamond indenter under a 50 g load for 15 s. The percentage change of SH (%SH) was calculated [% SH = 100 (SH_1_ - SH_0_)/SH_1_].

### Cross section hardness (CSH)

To perform CSH tests, the samples were sectioned perpendicularly to the surface through the center. One half of each sample was embedded in acrylic resin and polished as described before. Two rows of five indentations each were made, one at the center of the exposed dental enamel and another one at a distance of 100 μm from the central row of indentations using a 25-gram load for 15 s. The indentations were made at 25, 50, 75, 90, and 150 μm from the outer enamel surface. The mean values of the two measuring points were calculated at each distance from the surface. The Knoop microhardness number was converted to % volume mineral vs. depth, following the methods described by Featherstone, et al.^6^ (1983). Thus, this formula (v% = 4.3 √325+11.3) was used in this study to evaluate the change depth of enamel^6^.

### Statistical analysis

The data were analyzed using Statgraphics Centurion XVI software (STATPOINT Technologies, Inc, USA). Initially, all the data (SH_0_, SH_1_) were checked by Shapiro-Wilk's test and Levene's test. Based on these preliminary analyses, the SH_0_ and SH_1_ data were submitted to the one-way analysis of variance and the Tukey's HSD *post hoc* test. The % volume mineral data were analyzed by Kruskal Wallis and Mann-Whitney tests. All analyses were performed at a significance level of a=0.05. The graph was prepared by Graphpad prism 6.0 (GraphPad Software, Inc, USA).

## Results

From the initial 56 sections in this study, after pH-cycling we rejected three of them because of extensive loss of the external surface of the lesion, which made it too difficult for us to take measurements. The surface microhardness analysis of all groups is shown descriptively in Table 1. The specimens showed a significant decrease in microhardness after treatment (p<0.05). One-way ANOVA and Tukey's HSD *post hoc* test showed a statistically significant difference in the mean of enamel SH between the groups (p<0.05). The SH values observed for G3 (257±70), G2 (208±84) and G1 (205±70) groups differed from G4 (98±110). When comparing the G1 experimental group, the combined G2 and G3 treatment groups, we found no statistically significant difference among them (p<0.05). On the other hand, the combined treatment groups, both containing CPP-ACP, were statistically different from the artificial saliva group (p<0.05). There were no differences between G1xG2, G2xG3 and G1xG3 for demineralization inhibition (p>0.05).

**Table 1:** Surface microhardness results (mean±SD) of human enamel specimens according to different groups

Groups*	SH_0_ (Baseline)	SH_1_ (after treatment)	% SH (after treatment)
	Mean±S.D	Mean±SD	Mean±S.D
G1	315±38^a^	205±70^D^	-33±28^B^
G2	304±35^a^	208±84B^D^	-30±32^B^
G3	321±39^a^	257±70^B^	-42±73^B^
G4	328±27^a^	98±110^A^	-69±32^A^

Different upper and lower case superscript letters indicate significant difference between the tested groups at p<0.05. We use lower case superscript letters to compare the values of the same row and upper case letters to compare the values of each column.

* G1 (n=14)and G2, G3 and G4 (n=13)

Results showed statistical significance for the group and distance factors, which indicates that the effect of the treatments was different depending on the depth of the enamel surface.

**Figure 3:**
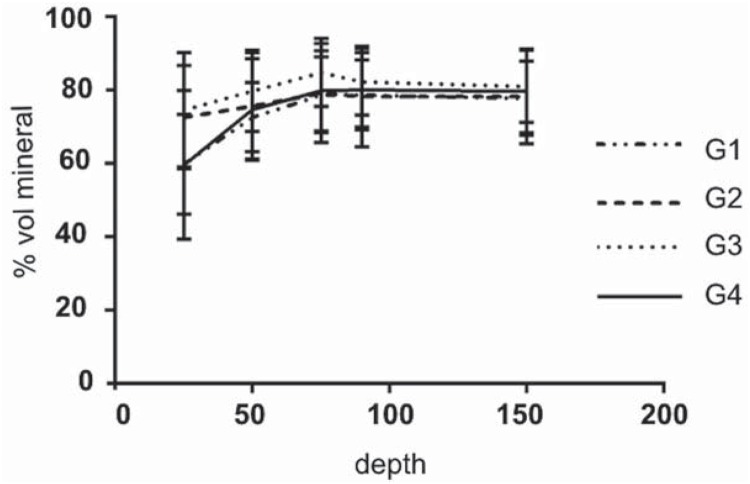
Mineral profile in the groups (G1, G2, G3 and G4)

The percentage of volume mineral vs. depth of the surface for all groups is shown in Figure 3. Percentage of volume mineral was statically significant in G3 when compared to the groups G1 and G4 (only at 25 and 50 pm). None of the groups differed significantly at other distances from the surface (p>0.05).

## Discussion

The objective of this study was to evaluate the effect of CPP-ACP cream with and without fluoride when associated with regular fluoride dentifrice on demineralized enamel using surface hardness and cross section hardness in a pH-cycling model, simulating oral cavity conditions. When carried out this way, the hardness measurement is very sensitive to changes in mineral density^2^. We converted the indentation lengths to % volume mineral according to Featherstone, et al.^6^ (1983).

In the cyclic model used in this study, we changed demineralization and remineralization solutions after each cycle, so that the concentration of calcium and phosphate ions in the solutions would not affect the results. The sub-saturation condition can lead to the dissolution of hydroxyapatite and diffusion of calcium and phosphate ions towards the enamel surface, reducing the SH after pH cycling^24^. In addition, we rinsed enamel surfaces after treatment with dentifrices, so that any treatment effect would be due to the binding of active ingredients to enamel^22^. Enamel demineralization was significantly reduced under these conditions and showed a significant difference when treatment was employed (Table 1).

When comparing G1 (FD 1,100 ppm F) to the combined groups (G2 and G3), we found no statistically significant difference among them (*p*<0.05). On the other hand, the combined treatment group and the FD group were statistically different from the artificial saliva group (*p*<0.05). Presumably, the concentration of fluoride decreased the porosity and solubility of enamel during exposure to acid challenge. Additionally, the specimens of the control group were exposed to the remineralizing solution without fluoride, which could decrease the remineralizing potential in the specimens of this group. Thus, this may explain the dose response of all groups.

We expected that the MPP cream comprising 900 ppm F and 1,100 ppm F of fluoride dentifrice would show a superior effect. However, the effect of treatment with MPP and FD was similar to the one only with FD. It is worth to highlight that we applied MPP and MP as a top coating, which could show a lower penetration to enamel surface compared to the NaF solution^7^. In contrast, we diluted the fluoride dentifrice to simulate the amount during brushing, so the uptake and fluoride reactivity are larger. These two aspects could have contributed to the results. Despite that, the G3 showed higher SH than the other groups (p<0.05). According to Pignatelli, et al.^15^ (2016), the application of NaF + CPP-ACP treatments can be synergistic, because while the former precipitates quickly, the latter forms a protective layer that involves the soluble NaF deposit readily, thus we expect “synergistic” benefits from it.

SH values obtained after using fluoride dentifrice and fluoride dentifrice associated with CPP-ACP products differed from those observed with saliva (G4 -negative control). The regular dentifrice associated with MPP showed better, but not statistically different results compared to MP. Our results suggest that both fluoridated dentifrice alone or combined with CPP-ACP were effective in preventing mineral loss. The results corroborate with other previous *in vitro* studies^10^ ^,^ ^16^. In those, authors found no significant difference between NaF 1,100 ppm and the application of combined groups^10^,^16^. Pulido, et al.^16^ (2008) suggested that a longer CPP-ACP application time can be necessary to remineralization. In another study, the CPP-ACP showed higher remineralizing potential when used with fluoridated toothpaste than when used alone. These authors reported that the MP was superior compared to the dentifrice with fluoride 1,100 ppm, despite not showing statistically significant difference^10^. Here, the time and application and the pH cyclic model were different when compared to the studies cited above, but relatively were similar.

Although there are few *in vitro* study evaluating the association of dentifrice with MP and MPP, our data corroborates a clinical report indicating that brushing with fluoride toothpaste and the application of CPP-ACP containing cream induced a significantly lower demineralization compared to the group undergoing no treatment, while CPP-ACP containing cream without fluoride showed little significant contribution to remineralization^11^. Clinically, Sitthisettapong, et al.^20^,^21^ (2015, 2012) did not detect any difference between the daily application of CPP-ACP containing cream immediately after brushing with fluoridated toothpaste and brushing with fluoridated toothpaste alone in nursery schools for a study period of one year. According to these authors, this might be due to the strong positive effects of fluoridated toothpaste, as observed in this study.

We aimed at evaluating the association between fluoride dentifrice and MP and MPP, not using them alone. In a recent publication by our research group using the same methodology, the microhardness values obtained after using MP and MPP formulations did not differ from each other, however, we obtained a response dose with MPP and FD^14^.

Microhardness values obtained after using MP (G2) and MPP (G3) and FD formulations did not differ from each other. These findings showed that there was no difference in the enamel surface microhardness, based on treatments with MP and MPP cream and fluoride dentifrice during cariogenic challenges, which can be due to the possibility of the fluoride ion in CPP-ACPF interacting with the ACP of the casein complex, rendering both inorganic components ineffective, or due to medium saturation^7^,^8^. The outcome measures for MI Paste Plus compared to MI Paste group indicates that the addition of fluoride does enhance remineralization^14^.

The surface hardness test (SH) is sensitive to detect the early stages of enamel demineralization at the enamel surface, but is not able to measure lesion depth^6^. Thus, we used cross section hardness (CSH) to evaluate the lesion depth^6^. Regarding the percentage of volume mineral vs depth, we observed that the combination MP and MPP and FD at 50 µ from the margin presented a successful dose response after five d of pH cycling. In this model, G1 produced a minor inhibitory effect on dental enamel lesion formation; in addition, this effect was not significantly different from the control group (G4). This study used 1,100 ppm fluoridated toothpaste three times a day for 1 m and the application of CPP-ACP cream once a day for 3 m. Figure 3 shows greater percentage of volume mineral from 25 to 75 pm in G3 group. Similarly, Pulido, et al.^16^ (2008) and Kumar, et al.^10^ (2008) observed, although not statistically different, the combined treatment group had a minor increase in lesion size when compared to the fluoride 1,100 ppm alone. These results and of the present study were consistent with an in vitro study by Mielczarek, et al.^12^ (2015) who observed significant enamel rehardness when the CPP-ACPF plus fluoride dentifrice (1,450 ppm F) was used. In the current study, the MP and MPP creams containing 10% (w/w) CPP-ACP had the same concentration used in other studies^5^ ^,^ ^7^ ^,^ ^12^ ^−^ ^14^ ^,^ ^16^ ^,^ ^19^ ^,^ ^22^ ^,^ ^23^.

Clinically, Shen, et al.^19^ (2011) demonstrated that there is need for an additional remineralization by extrinsic calcium, phosphate, and fluoride ions to increase the natural remineralization of saliva. These authors showed that MI Paste and MI Paste Plus provided high levels of salivary calcium and phosphate and enhanced remineralization of enamel lesions more than other products with high concentrations of fluoride. Previously, Renolds, et al.^18^ (2008) observed that the dentifrice containing 2% CPP-ACP and 1400 ppm F was better than all other formulations. Microradiography of the remineralized lesions demonstrated that fluoride dentifrices (1,100 ppm F and 2800 ppm F) remineralized predominantly at the surface, whereas the 2% CPP-ACP dentifrice and the 2% CPP-ACP with 1400 ppm F dentifrice produced a more homogenous remineralization throughout the lesion body. Thus, the exposure of the specimen to intra-oral conditions leads to key factors such as product dilution with saliva, product adsorption into soft and hard surfaces, and clearance. Thus, the Tooth Mousse Plus product provided high concentrations of stabilized and bioavailable calcium, phosphate, and fluoride ions in saliva and enhanced remineralization of *in situ* enamel subsurface lesions compared to products only containing fluoride^19^.

In summary, the findings of the this study suggest that the MP and MPP, when associated with fluoride toothpaste, under laboratorial conditions, were able to inhibit carious lesions in enamel and showed no difference compared to the use of fluoride dentifrice alone. However, prevention of lesion depth was better when we used the MPP and fluoride dentifrice together. Despite the limitations of this *in vitro* study, the use of regular fluoride dentifrice and agents based on calcium and phosphate and fluoride compounds could be useful to control carious lesions.

## Conclusion

The results obtained in this study showed similar remineralizing effects for MI Paste and MI PASTE Plus with fluoride dentifrice on enamel early lesions. MI PASTE Plus with fluoride dentifrice showed greater reduction in lesion depth.
